# Only Connect: The Functional Architecture of Brain Connectivity

**DOI:** 10.1371/journal.pbio.0020411

**Published:** 2004-10-26

Imagine three cities, A, B, and C, splayed across the landscape to form a triangle, with each connected to the other two by two-lane roads. Such an arrangement of cities and roads constitutes a structural network. On any given day traffic may flow, say, only from A to B to C, or in both directions between A and B but from C only to A, or in both directions between all three, or any one of ten other arrangements. Within this structural network, then, there are 13 possible functional networks. If these cities are embedded within a larger network of routes and destinations, their particular triangular traffic pattern represents a “motif” of connectivity, akin to a recurring musical motif within a larger symphony.

Such connectivity networks are central to information processing in the brain, and understanding the recurring structural and functional motifs they contain is one way to begin to dissect how the symphony of brain function is composed. In this issue, Olaf Sporns and Rolf Kötter identify several common motifs in real brain networks, and show that brains tend to maximize the number of functional motifs while keeping the number of structural motifs relatively low.

**Figure 1:**
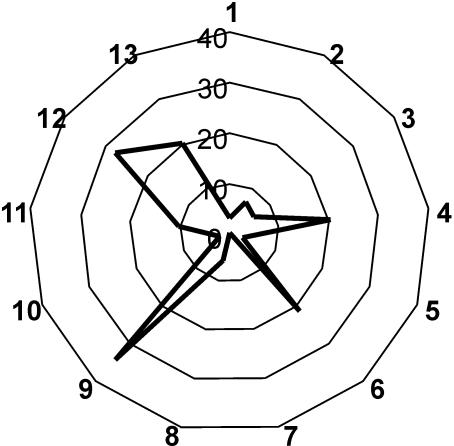


The authors began with the frequency of motifs of different sizes (two, three, four, or five nodes) found in the visual cortex and whole cortex of the macaque monkey, the cat cortex, and the nervous system of the nematode Caenorhabditis elegans. For comparison, they generated matrices that contained an equivalent number of components (nodes and connections), but whose connections were either random or lattice-like, in which all nearest neighbors were connected. They found that, compared to the artificial networks, the biological ones were relatively low in structural diversity. For instance, macaque visual cortex contained instances of 3,697 different motifs with five nodes, versus 8,887 for equivalent random networks. Functionally, however, unlike the artificial systems, the biological systems were maximally diverse, with the maximum functional motif diversity (e.g., 13 for three vertices and 9,364 for five vertices) observed in all motif sizes they investigated.

The researchers also found some intriguing patterns within this maze of connectivity. For instance, not all motifs were found in equal numbers. A common functional motif for three vertices was for both A and C to communicate back and forth with B, but not with each other. This structure allows B to function as an integrator of signals from A and C, while keeping the activities of A and C distinct from one another. This kind of structure is widespread throughout the nervous system.

The authors then ran an evolutionary algorithm on their artificial networks. They showed that by selecting for maximal functional motif number, the structure of the artificial systems quickly came to resemble the structure of the real ones, with dense local connections and relatively fewer long-distance ones. Such a structure, termed “small world” connectivity, promotes cooperation between functional units, and efficient information exchange. Taken together, these results suggest that one factor that may drive the evolution of neural architecture is the maximization of functional connectivity within a network of relatively few neural actors.

